# Supplementary material for: cGAS-STING dependent type I IFN protects against Leptospira interrogans renal colonization in mice
Source: bioRxiv. 2025 Jun 4:2025.06.02.657349. Preprint. [Version 1] doi: 10.1101/2025.06.02.657349 (PMC12157660; doi:10.1101/2025.06.02.657349)
Supplement: 1 [file NIHPP2025.06.02.657349v1-supplement-1.pdf]

bioRxiv preprint doi: https://doi.org/10.1101/2025.06.02.657349; this version posted June 4, 2025. The copyright holder for this preprint (which was not certified by peer review) is the author/funder, who has granted bioRxiv a license to display the preprint in perpetuity. It is made available under aCC-BY 4.0 International license.

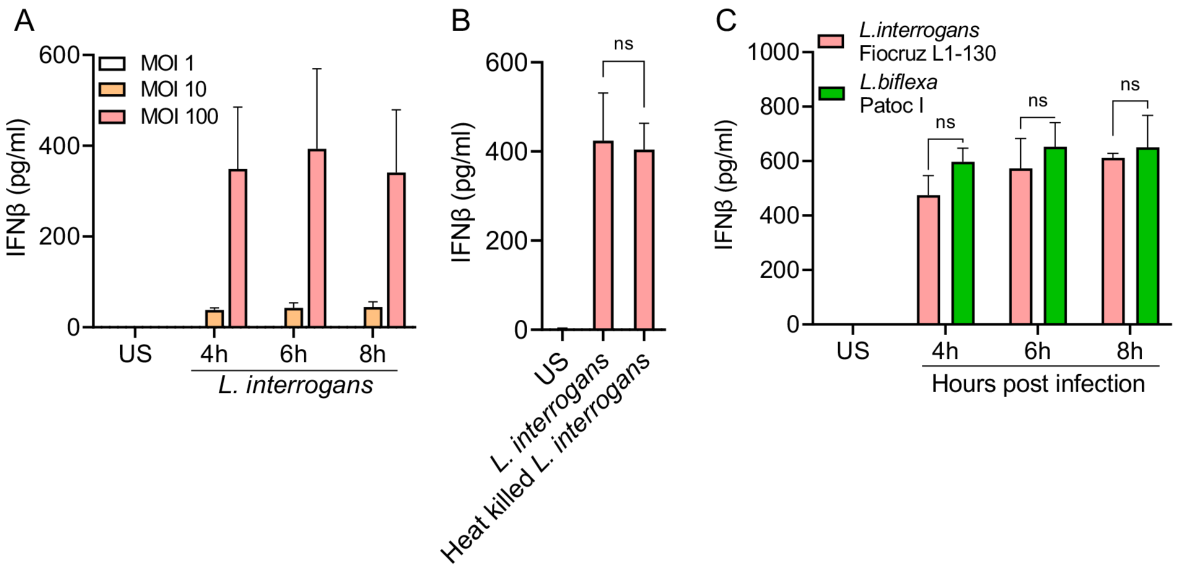

S1 Fig. Analysis of IFNβ production from BMDM infected with *Leptospira.* (A) Dose dependent induction of IFNβ was quantified by ELISA in culture supernatants of WT BMDMs infected with *L. interrogans* (MOI 1, 10, 100) for 4, 6, and 8h. Data are pooled from two independent experiments and expressed as the mean ± SD. (B) Quantification of IFNβ levels by ELISA in culture supernatants of WT BMDM infected with live or heat-killed *L. interrogans* (MOI 100) at 6h post-infection. Data are pooled from three independent experiments. Statistical significance was calculated by unpaired Student's *t* test. (C) Quantification of IFNβ levels by ELISA in culture supernatants of WT BMDM infected with *L. interrogans* Fiocruz L1-130 (MOI 100) or *L. biflexa* Patoc 1 (MOI 100) at 4, 6, and 8h post-infection. Statistical significance calculated by two-way ANOVA. ns=non-significant.

bioRxiv preprint doi: https://doi.org/10.1101/2025.06.02.657349; this version posted June 4, 2025. The copyright holder for this preprint (which was not certified by peer review) is the author/funder, who has granted bioRxiv a license to display the preprint in perpetuity. It is made available under aCC-BY 4.0 International license.

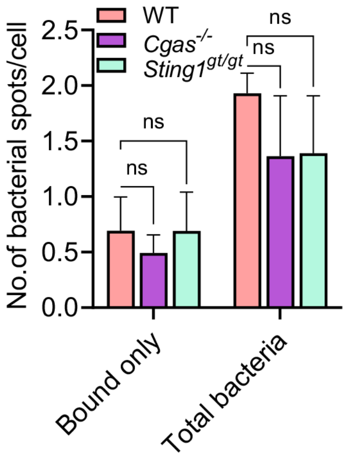

S2 Fig. Analysis of *L. interrogans* binding and internalization by BMDM. Quantification of immunofluorescence images of bacteria bound or total bacteria (bound and internalized) in WT, *Cgas*$^{-/-}$ and *Sting1*$^{gt/gt}$ BMDMs at 3h post infection with *L. interrogans* (MOI 100). Data are pooled from three frames per experiment from three independent experiments. Statistical significance was calculated by two-way ANOVA. ns=non-significant.

bioRxiv preprint doi: https://doi.org/10.1101/2025.06.02.657349; this version posted June 4, 2025. The copyright holder for this preprint (which was not certified by peer review) is the author/funder, who has granted bioRxiv a license to display the preprint in perpetuity. It is made available under aCC-BY 4.0 International license.

S1 Table: Primers used for qPCR

**Mouse Primers**	**Sequence (5'-3')**
Ifna (F)	CCT GAG AGA GAA GAA ACA CAG CC
Ifna (R)	GGC TCT CCA GAC TTC TGC TCT G
Ifnb (F)	GCT CCT GGA GCA GCT GAA TG
Ifnb (R)	CGT CAT CTC CAT AGG GAT CTT GA
Ifit 1 (F)	CAA GGC AGG TTT CTG AGG AG
Ifit 1 (R)	GAC CTG GTC ACC ATC AGC AT
Ifit 3 (F)	TTC CCA GCA GCA CAG AAA C
Ifit 3 (R)	AAA TTC CAG GTG AAA TGG CA
Ifi 44 (F)	CTG ATT ACA AAA GAA GAC ATG ACA
Ifi 44 (R)	AGG CAA AAC CAA AGA CTC CA
Zbp 1 (F)	TCA AAG GGT GAA GTC ATG GA
Zbp 1 (R)	GTG GAG TGG CTT CAG AGC TT
Tnfa (F)	GGT GCC TAT GTC TCA GCC TCT T
Tnfa (R)	GCC ATA GAA CTG ATG AGA GGG AG
Ppia (F)	GAG CCA CTC ACC TGA TGC TTA
Ppia (R)	GGC AAT GAA AAT GCT ACC ACC TT
Actin b (F)	CGA GGT ATC CTG ACC CTG AA
Actin b (R)	GGT GTG GTG CCA GAT CTT CT
**Human primers**	**Sequence (5'-3')**
Ifnb (F)	AAA CTC ATG AGC AGT CTG CA
Ifnb (R)	AGG AGA TCT TCA GTT TCG GAG G
Actin b (F)	CAC CAT TGG CAA TGA GCG GTT C
Actin b (R)	AGG TCT TTG CGG ATG TCC ACG T
**Bacteria primers**	**Sequence (5'-3')**
lipL32 (F)	AAG CAT TAC CGC TTG TGG TG
lipL32 (R)	GAA CTC CCA TTT CAG CGA TT
